# “It’s More than Just Exercise”: Psychosocial Experiences of Women in the Conscious 9 Months Specifically Designed Prenatal Exercise Programme—A Qualitative Study

**DOI:** 10.3390/healthcare13070727

**Published:** 2025-03-25

**Authors:** Beata Makaruk, Weronika Grantham, Wanda Forczek-Karkosz, Maciej Płaszewski

**Affiliations:** 1 Department of Physical Education Methodology, Faculty of Physical Education and Health in Biała Podlaska, Józef Piłsudski University of Physical Education in Warsaw, 00-968 Warsaw, Poland; beata.makaruk@awf.edu.pl; 2 Department of Dance, Faculty of Physical Education and Health in Biała Podlaska, Józef Piłsudski University of Physical Education in Warsaw, 00-968 Warsaw, Poland; 3 Department of Biomechanics, Faculty of Physical Education and Sport, University of Physical Education in Krakow, 31-571 Krakow, Poland; wanda.forczek@awf.krakow.pl; 4 Institute of the Principles of Physiotherapy, Faculty of Physical Education and Health in Biała Podlaska, Józef Piłsudski University of Physical Education in Warsaw, 00-968 Warsaw, Poland; maciej.plaszewski@awf.edu.pl

**Keywords:** pregnancy, exercise, prenatal physical activity, social support, group exercise, adherence, personal experiences, holistic programmes

## Abstract

**Background/Objectives**: Physical activity during pregnancy is recognised for its benefits to maternal and foetal health. However, adherence to prenatal exercise programmes is often low due to psychological, physical, and social barriers. This study explored the experiences of women who successfully completed a tailored prenatal exercise programme, “Conscious 9 Months”, aiming to identify factors enabling adherence and providing recommendations for future programme development. **Methods**: A descriptive qualitative research design was employed using semi-structured, in-depth interviews with ten participants who completed the programme between 2017 and 2019. Thematic Analysis was conducted to explore their experiences. **Results**: Participants emphasised the transformative nature of the programme, which extended far beyond physical exercise. Three interconnected domains emerged: psychosocial, physical, and psychoeducational. The present article focuses on the psychosocial domain, highlighting the significance of exercising in a group of pregnant women as a key source of support. Additionally, the atmosphere played a crucial role in facilitating the participants’ regular attendance. Finally, the findings indicate that the programme fostered lasting social connections that extended beyond its formal completion. **Conclusions**: A holistic approach that integrates physical activity with social and emotional support can foster a sense of community and enhance long-term adherence. The design of future programmes should prioritise small group sizes to facilitate peer support and create a safe and welcoming atmosphere to encourage emotional expression. Additionally, sustained engagement beyond pregnancy through follow-up initiatives, family involvement, and postnatal movement programmes can further reinforce long-term participation and promote lasting health benefits for both mothers and their children.

## 1. Introduction

Physical activity during pregnancy is recognised as a key factor in supporting the health of both the mother and the developing foetus [1,2,3,4,5,6,7]. Studies have consistently shown that regular exercise improves the physical condition of pregnant women, reduces the risk of pregnancy complications, and supports mental and emotional health, while also preparing the body for childbirth and the postpartum period [8,9,10,11,12,13,14]. Regular physical activity during pregnancy has a beneficial impact not only on the physical condition and overall well-being of the mother [15] but also on the baby’s health. In an uncomplicated pregnancy, exercise is safe for both the mother and the foetus, supporting optimal foetal development by enhancing uterine blood flow and ensuring a safe intrauterine environment [7,10,11,16].

The current guidelines of the American College of Obstetricians and Gynaecologists (ACOG) recommend that pregnant women engage in at least 30 min of moderate-intensity physical activity on most days of the week [10]. Similarly, the Canadian Society for Exercise Physiology (CSEP) and Society of Obstetricians and Gynaecologists of Canada (SOGC) recommend that pregnant women accumulate at least 150 min of moderate-intensity physical activity each week to achieve clinically meaningful health benefits and reductions in pregnancy complications [17]. Likewise, the Polish Society of Gynaecologists and Obstetricians (PTGiP) and Polish Society of Sports Medicine (PTMS) recommend that women with uncomplicated pregnancies engage in at least 150 min of moderate-intensity physical activity per week throughout the entire pregnancy [18]. These guidelines emphasise the importance of individualised approaches, elimination of high-risk activities, and support for pregnant women, including those who were previously inactive, in engaging in safe forms of exercise [19,20,21].

Recommended forms of physical activity can include a variety of exercises with unique benefits, ranging from improved flexibility and relaxation to strengthening muscles and enhancing cardiovascular endurance, allowing the exercise programme to be tailored to the individual needs of expectant mothers [20,22,23,24,25,26].

Despite such widespread and consistent guidelines and recommendations, strong and widely disseminated evidence on the benefits of prenatal physical activity, its implementation remains a persistent problem, and many women encounter difficulties in maintaining it at an appropriate level [27,28,29]. Pregnancy, often seen as a “teachable moment” for adopting healthier behaviours, paradoxically sees a decline in physical activity levels [30]. Physical activity during pregnancy remains significantly low, as reflected in data from various countries. In the USA, only 12.7–45% of pregnant women meet ACOG recommendations for moderate physical activity, while in Norway, only 25% (in the 28th week of pregnancy) [31], and in Canada, this figure is just 23%, compared to 33.6% of non-pregnant women [29,32]. In Poland, the situation is equally concerning: over 60% of women reduce their physical activity early in pregnancy, and only 25% meet the minimum recommendation of 150 min of moderate activity per week [33].

Adherence to recommended exercise programmes during pregnancy presents a significant challenge, as many women who initiate physical activity discontinue it before term. Yeo et al. [34] found that previously sedentary pregnant women exhibited varied adherence patterns, with many reducing or ceasing exercise as pregnancy progressed. Similarly, Nascimento et al. [26] reported that only 20.1% of pregnant women maintained physical activity throughout their pregnancy, with adherence declining most notably in the first and third trimesters. However, research directly comparing physical activity patterns across different exercise programmes remains limited, constraining conclusions about adherence to physical activity during pregnancy [35,36].

In this article, the terms adherence, participation, and engagement are used in different contexts to reflect the multifaceted nature of prenatal physical activity within the holistic care framework. Adherence refers to the extent to which women consistently follow the recommended exercise programme, highlighting commitment and continuity, which has been shown to be influenced by factors such as social support and programme structure [37]. Participation denotes the act of attending and taking part in exercise sessions, capturing the practical aspect of involvement, as seen in studies demonstrating that group-based settings enhance attendance rates [38]. Engagement encompasses a broader, more active involvement, including emotional, social, and psychological investment in the programme, which extends beyond mere attendance, aligning with findings that highlight the role of group dynamics and trainer support in fostering motivation and long-term behavioural change [39,40].

These data highlight the scale of the challenge and underscore the need for a deeper understanding not only of the barriers but also of the facilitators that help pregnant women maintain the recommended physical activity throughout pregnancy. Such insights are essential for promoting regular and safe physical activity as a key component of maternal and child health [29].

Existing research, including qualitative studies, highlights the complex interplay of barriers and enablers that shape women’s experiences of exercising during pregnancy. Among the most common barriers cited are pregnancy-related symptoms such as fatigue, nausea, and physical discomfort, which often limit activity to walking or performing household tasks [21,33,41,42,43,44]. Psychological concerns, particularly fear of harming the baby or uncertainty about safe exercise practices, further contribute to a significant decline in physical activity levels. Additionally, social and cultural factors, including lack of support from family or healthcare providers and societal expectations, may discourage participation in exercise during pregnancy [27,28]. Many non-exercising women also report a lack of knowledge, perceived risks, or conflicting information, which further reinforces their inactivity [27].

Despite these barriers, several key facilitators have been identified that help women maintain physical activity during pregnancy. Women who engage in regular exercise often report emotional well-being, a sense of accomplishment, and preparation for childbirth as strong motivators [45]. Social support—whether from family, peers, or healthcare professionals—plays a crucial role in fostering confidence and motivation [43]. Structured group exercise programmes are particularly beneficial, providing professional guidance, social connections, and emotional reinforcement, which help reduce feelings of isolation and improve adherence [44]. Participation in walking groups or structured exercise classes has also been shown to enhance the sense of community and belonging, further encouraging engagement in physical activity [28].

While various barriers to prenatal physical activity have been identified and some studies have explored enablers, a notable knowledge gap remains regarding the lived experiences of pregnant women who successfully maintain the recommended activity levels. Studies on prenatal physical activity focus primarily on analysing the technical parameters of exercise programmes, such as intensity, type of activity, or their impact on physical health [23,46,47,48], often overlooking the personal narratives and strategies of those who adhered to physical activity guidelines. Previous studies focusing on lived experiences connected to prenatal exercise have explored various aspects of this engagement, including lifestyle choices, social experiences and decision-making processes. For instance, Newson et al. [49] investigated women’s opinions and lived experiences related to maintaining a healthy lifestyle, gestational weight gain, and physical activity during pregnancy. Livingston et al. [50] examined how pregnant women made sense of their physical activity-related social experiences as pregnancy progresses. Findley et al. [51] explored women’s experiences and decision-making about physical activity during and after pregnancy.

However, there remains a significant gap in understanding the implications for practice, that is, how to support women in maintaining the recommended levels of physical activity during this period of life. Such support from professionals through proper training and personalised care is essential [28,30,52].

Thus, in order to better understand the experiences of women who successfully maintained the recommended exercise levels during pregnancy, it is necessary to take a wider view—not only of the physical but also of the social, psychological, and subjective areas. This is possible by applying qualitative research methodologies to explore the experiences and opinions of women who directly face the challenges of physical activity during pregnancy [41]. This period is a unique time in a woman’s life, characterised by complex changes that go beyond physiology, encompassing subtle aspects of their identity, emotions, and social relationships [53]. This is an important context in the field of prenatal physical activity, as it highlights the distinct needs of women during this life stage, with distinct experiences. Exploring the lived experiences of pregnant women regarding exercise is crucial for understanding the complex factors that influence their physical activity behaviours during pregnancy. Qualitative research methods provide rich contextual insights into women’s personal perceptions, motivations, and challenges related to prenatal exercise.

Between 2017 and 2019, the first author of this study, an exercise specialist who led the exercise sessions for pregnant women, designed and conducted a comprehensive programme of regular physical activity titled “Conscious 9 Months”. During this period, she observed remarkably high adherence among participants who consistently attended classes throughout their pregnancies. This observation sharply contrasted with the declining trends described in the existing literature and motivated a qualitative study to explore the experiences of women who completed the entire programme and exercised regularly.

This study aims to examine the participants’ experiences and to identify and analyse the key factors and conditions that enabled them to remain physically active. The innovation of this study lies in its focus on women who successfully maintained regular exercise and participated in the entire “Conscious 9 months” programme, providing insight into the factors that supported their adherence [34]. Additionally, the study seeks to explore the specific aspects of the programme that participants found particularly important, helpful, and motivating, in order to inform the future design of successful prenatal exercise programmes.

During data analysis, it became evident that the participants’ experiences extended beyond physical exercise, encompassing psychosocial and psychoeducational domains that significantly contributed to their adherence. Given the depth and complexity of the findings, the results are reported in three separate but interconnected articles, each focusing on one of the following domains: psychosocial, physical, and psychoeducational. This approach allows for a more detailed and nuanced exploration of each aspect while improving clarity for the reader. The present article focuses on the psychosocial domain, which participants identified as the most crucial factor in their regular attendance. The second article will explore the physical domain, examining the programme’s structure, flexibility, the approach to exercises, and the role of the trainer in providing personalised support. The third article will address the psychoeducational domain, focusing on how the programme reduced fear and anxiety around pregnancy and childbirth while equipping women with valuable knowledge. While the three domains are presented separately for analytical clarity, they are deeply interconnected, reinforcing one another and shaping the programme’s overall impact as a transformative experience.

## 2. Materials and Methods

### 2.1. Study Design

A descriptive qualitative research design with an inductive approach was used. This approach was chosen over other qualitative methodologies because it allows for a straightforward representation of participants’ experiences without imposing an interpretative framework, as seen in phenomenology, or seeking to develop a theory, as in grounded theory [54]. The descriptive qualitative approach enabled us to capture rich, detailed accounts of women’s perspectives while remaining close to their lived realities. Given the objectives of this study, which were to explore and analyse facilitators of adherence rather than generate abstract theoretical constructs, this methodology provided the necessary flexibility and depth to address our research questions effectively.

In-depth, individual, semi-structured interviews with thematic analysis (TA) [55] were used to address the study’s aims.

**Figure 1:**
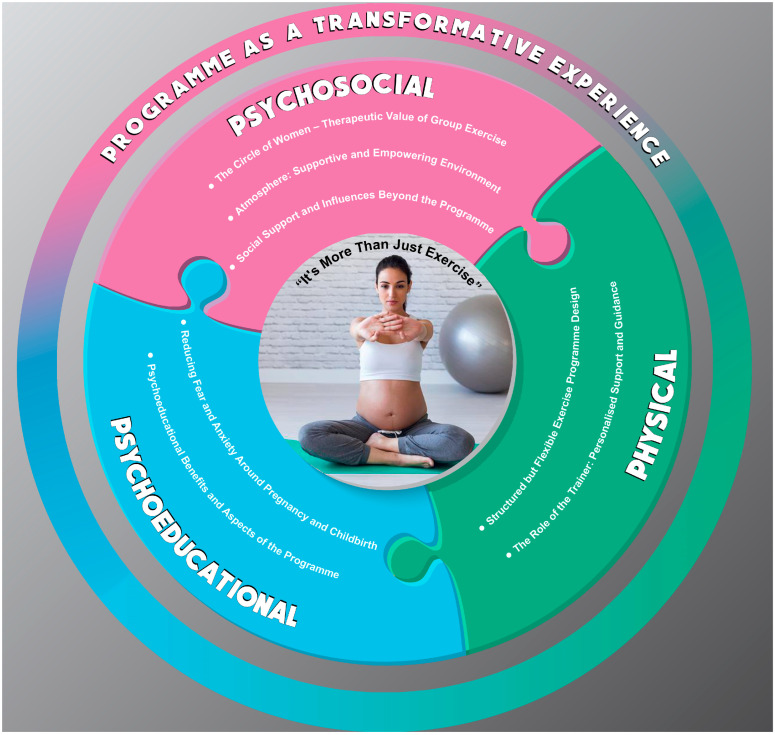
“It’s More than Just Exercise”—Programme as a Transformative Experience—Key Domains and Themes. The graphic illustrates the “Conscious 9 Months” programme, designed as a holistic prenatal physical activity intervention, integrating exercise, social and emotional support, and education.

This study adhered to the COREQ guidelines for reporting qualitative research [56]. Table S2 shows the COREQ checklist and the study’s flowchart corresponding to the timeline presented in Figure 1 of the published protocol for the current study [57]. This project was approved by the Senate Ethics Committee of the Józef Piłsudski University of Physical Education in Warsaw (No. SKE-2/2022) and followed the Declaration of Helsinki.

### 2.2. Intervention: The “Conscious 9 Months” Exercise Programme

The “Conscious 9 Months” is a regular exercise programme designed specifically for women experiencing physiological pregnancy. It was a blend of scientifically based training principles tailored to each expectant mother, taking into account her individual needs and expectations. The programme offered twice-weekly sessions, each lasting 60–90 min, and consisted of a warm-up, a main section, and a relaxation phase. The exercises were tailored to each trimester of pregnancy, ensuring safe support for physical fitness and preparation for childbirth. A key feature of the programme was its individualised approach, with small group sizes (6–8 participants), enabling personalised adjustments to meet participants’ unique physiological and emotional needs.

A detailed description of the programme can be found in the study protocol [57]. The programme described here was also used in a separate study designed to evaluate foetal safety, where a description of a sample session can be found [7].

### 2.3. Study Participants

Purposeful sampling was used in order to meet the study’s objectives. The participants were contacted via telephone by the research team in January and February 2022 because in the years 2017–2019, they completed the “Conscious 9 months” programme.

The study included women who completed the entire programme and participated throughout their pregnancies.

The study excluded women who could not participate in the programme due to medical reasons; i.e., the physiological state of pregnancy did not allow for participation and there was no medical clearance, as well as women who did not agree to have their interview recorded.

All participants were Polish and resided in the town of Biała Podlaska. Additional characteristics of the participants are presented in Table 1 and Table 2.

### 2.4. Data Collection

To address the research questions, face-to-face, in-depth, semi-structured individual interviews were conducted with ten research participants between 30 March 2022 and 26 April 2023 to collect qualitative data through detailed content and genuine conversations.

To recruit participants, all eligible women were initially contacted via telephone and invited to participate in the study. Ten out of twelve responded positively and were then sent an email containing detailed information about the study (including its aims, objectives, methods, and procedures) to enable them to make an informed decision. The remaining two participants could not be contacted by phone. Following their initial agreement, the ten women who agreed to participate were contacted to arrange a convenient date and time for a face-to-face interview. Written informed consent was obtained from all participants prior to data collection.

The data were collected as digital audio recordings using both a dictaphone and telephone. The interviews were manually transcribed verbatim and anonymised to ensure participant confidentiality. Additionally, the transcripts were returned to the participants for review, allowing them to provide comments and/or corrections.

#### In-Depth Interviews

To minimise the risk of bias, the interviews were not conducted by the first author, who had served as the participants’ trainer during the programme. Instead, a neutral interviewer—a qualified psychologist with experience working with pregnant women—was selected. The psychologist, who was unfamiliar to the participants, was of the same nationality (Polish) and conducted the interviews in the participants’ native language. She received instructions on qualitative research methods, including in-depth interviewing techniques. Participants were reassured that the study sought their authentic experiences, emphasising that there were no “correct” answers.

The interview guide (see Table S2, [57]) underwent pilot testing with a sample resembling the study participants. Following the pilot test interview, several refinements were made to the interview guide to enhance the data collection. Some questions were reworded to ensure that they were fully open-ended, encouraging participants to provide more descriptive and reflective responses. Additional introductory questions were incorporated at the beginning of the interview to help participants feel more comfortable and gradually ease into the main topic. Furthermore, the interviewer was instructed to slow the pace of the interview, allowing participants more time to formulate their answers before offering prompts or asking follow-up questions. These adjustments were implemented in all subsequent interviews to ensure a more natural and participant-led conversation flow. The script served as a flexible guide, with each participant’s responses shaping the flow of the conversation.

Efforts were made to create a safe and comfortable environment for the participants to openly share their experiences. Interviews were conducted in a private, quiet room at the university, located away from the main campus, to minimise disturbances. Scheduling was flexible and based on each participant’s preferences. Initial rapport-building questions were used to establish trust and to reduce tension. Participants were reminded that they could take their time, discuss topics important to them, or stop the interview at any point. The interviewer adopted a sensitive and open-ended approach to questioning.

Field notes were taken during the interviews to capture nonverbal cues, such as facial expressions, gestures, tone of voice, and emotional reactions (e.g., laughter or tears), which were incorporated into the transcriptions. At the end of each interview, the participants were invited to share any additional thoughts about their experiences.

Sociodemographic data were collected via a brief questionnaire, and member-checking procedures were employed to ensure the accuracy of the findings and their alignment with the participants’ perspectives.

Each interview lasted approximately 45–60 min.

The interview guide is described in detail in Table S2 of the study protocol [57].

### 2.5. Data Analysis

The transcribed interviews were analysed using a thematic analysis approach, as described by Braun and Clarke [55]. Thematic Analysis is a widely used qualitative method for identifying, analysing, and reporting patterns (themes) within data. It provides a flexible framework that can be adapted to suit a variety of research questions and epistemological approaches. The method involves six key phases: familiarisation with the data, generating initial codes, searching for themes, reviewing themes, defining and naming themes, and producing the final report. This approach emphasises researcher reflexivity and transparency in the analysis process, enabling a rich and detailed understanding of the participants’ experiences and perspectives. By systematically organising data into meaningful categories, Thematic Analysis facilitates the exploration of both explicit and implicit patterns, offering valuable insights into complex phenomena.

The analyses were conducted separately by the two authors (BM and WG), and in case of discrepancies, the third author (WF-K) was contacted for consultation. The step-by-step Thematic Analysis process of this study is presented in Table 3.

#### 2.5.1. Data Organisation and Management

In this study, we conducted manual coding without the use of qualitative data analysis software (such as NVivo, Atlas.ti), a practice that remains widely accepted in qualitative research [55]. Manual coding allowed for deep, immersive engagement with the data, facilitating a nuanced and reflexive approach to theme development.

To ensure rigour and organisation, transcripts were carefully structured and analysed using an iterative process. Data were systematically coded by two researchers, and coding sheets were maintained to track the development of themes.

#### 2.5.2. Reliability Checks

In order to maintain intercoder reliability, two researchers (BM, WK) independently coded the data, and regular coding consensus meetings were held to discuss and resolve any discrepancies. Coding disagreements were resolved through discussion, ensuring that the themes accurately reflected the participants’ experiences. In cases where consensus was not immediately reached, a third team member (WF-K) was consulted for an additional opinion.

To further enhance consistency and coherence, regular meetings were held throughout the analysis process to refine the coding framework and ensure alignment with the interpretation of themes. This iterative approach helped maintain analytical rigour and reliability, in line with the best practices in qualitative research [58,59].

#### 2.5.3. Data Saturation

An ongoing evaluation process was employed, and the interviews were pre-analysed concurrently with data collection. Saturation was considered reached when no new codes, insights, or significant variations in responses emerged, suggesting that additional interviews were unlikely to yield novel insights [60]. While we did not establish a strict a priori stopping rule, our decision to conduct 10 interviews was informed by existing qualitative research indicating that small, homogeneous participant groups, particularly in experience-focused studies, often achieve saturation within this range [61]. Additionally, our sample was highly specific, comprising only women who had participated in this particular prenatal exercise programme. Given the limited pool of eligible participants, our approach ensured that we captured a comprehensive range of experiences within this defined group.

To enhance the rigour of the analysis, peer debriefing was conducted with two research experts (WF-K, MP), and member-checking was employed to confirm the accuracy of the findings with the participants.

### 2.6. Trustworthiness

This study adopts a relativist approach to trustworthiness [62], where the quality of a study is not determined by a fixed set of criteria, as in the criteriological approach [63], but rather “is both revealed and resides in the research report” [62]. Thus, quality depends on the rigour of the researchers’ work and, equally importantly, on the response of the readers—their judgement and interpretation.

Additionally, the measures ensuring trustworthiness in this study are based on the framework proposed by Smith and Caddick [64] and include substantive contribution, impact, width, coherence, credibility, and transparency.

The substantive contribution is demonstrated through the study’s ability to deepen the understanding of pregnancy exercise programmes, offering both scientific insights and a clear textual structure that supports broader interpretations.

Impact is achieved by engaging the reader, prompting critical reflection, and potentially inspiring new practices or further research in this field.

Width is ensured through comprehensive data collection, including in-depth interviews and a rigorous interpretive process, supported by participant quotations and consideration of alternative explanations.

Coherence is maintained by aligning the findings with existing research and theories and presenting an internally consistent and externally relevant analysis.

Credibility is enhanced through member-checking procedures, which enable participants to validate interpretations and contribute to a nuanced understanding of the data.

Transparency is strengthened through collaboration with co-authors from diverse disciplines (WF-K, MP), who acted as critical friends. By examining theoretical preferences and scrutinising assumptions, we contributed to a balanced and robust analysis, ensuring that the findings reflect an open and reflexive research process.

## 3. Results

The 10 participants were between 34 and 44 years old, and their educational levels ranged from secondary vocational to higher education (seven with university degrees and three with secondary vocational education). Nine participants were in their first pregnancy, and one was in her second pregnancy. Only three of the participants were physically active before becoming pregnant, regularly engaging in various forms of physical activity, while seven did not engage in regular exercise before pregnancy (see Table 1). They also differed in terms of professional activity during pregnancy, with eight women opting for sick leave, mostly between the 9th and 37th weeks, while one woman remained active at work until the 38th week of pregnancy. The study began for most women between the 6th and 20th weeks of pregnancy (see Table 2).

The overarching theme emerging from the interviews was titled “*It’s More than Just Exercise*”, reflecting the holistic nature of participation in the programme. To explore this theme, the findings were organised into three interconnected domains: psychosocial, physical, and psychoeducational, as shown in Figure 1, depicting the entire programme as a transformative experience for the women. The three domains are presented as three separate articles, each focusing on one domain.

Three key domains contributed to its transformative impact, resulting in participants feeling that the programme was “more than just exercise”. The entire programme is explored and analysed in three separate articles, each focusing on one domain:

- Psychosocial domain—Small group sessions, consisting of women at different pregnancy stages, fostered a supportive community, reducing feelings of isolation and enhancing a sense of empowerment among participants. The shared pregnancy experience and the therapeutic value of group exercise enhanced motivation and encouraged adherence. The programme was characterised by a warm, supportive atmosphere, where emotions could be freely expressed.
- Physical domain—The programme featured structured yet flexible training sessions tailored to the individual needs of each pregnant woman. The trainer played a crucial role in providing personalised support, adapting exercises to participants’ physical capabilities, and fostering a sense of support and connection.
- Psychoeducational domain—The sessions reduced anxiety surrounding childbirth and equipped participants with knowledge about bodily changes, breathing techniques, birth, and the postpartum period. Education was seamlessly integrated into the movement, ensuring that women not only engaged in physical activity but also actively prepared for labour and motherhood.

Thus, the “Conscious 9 Months” programme was not just about exercise but a comprehensive system of social, physical, emotional, and educational support, ensuring holistic preparation for childbirth and early motherhood, where participants felt cared for and motivated to attend regularly.

**Figure 2:**
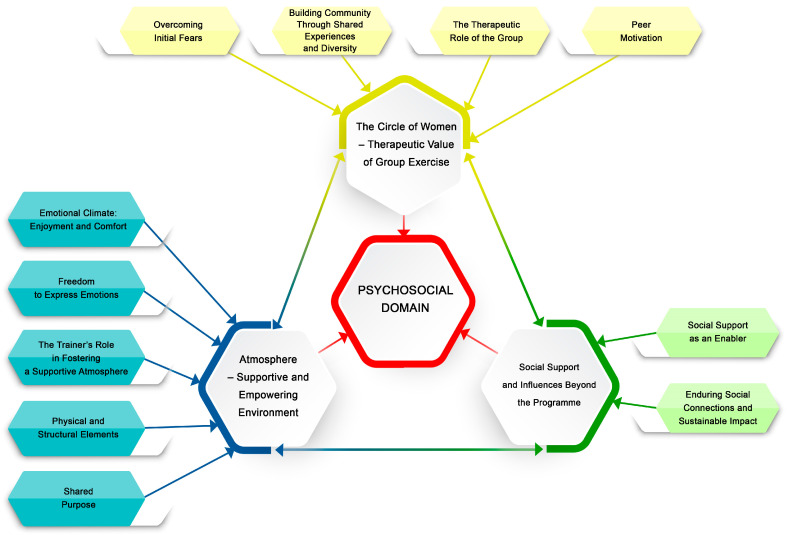
Thematic map depicting the main themes and sub-themes in the psychosocial domain of the programme.

The present article examines the psychosocial domain, which was identified as the most crucial by the participants. When presenting the results, themes, and sub-themes, more illustrative quotes can be found in Table S1. The main themes explored in this article include *The Circle of Women: Therapeutic Value of Group Exercise*, *Atmosphere: Supportive and Empowering Environment*, and *Social Support and Influences Beyond the Programme.* A thematic map depicting the main themes and sub-themes of this study is shown in Figure 2.

One of the key domains of the “Conscious 9 Months” programme, namely the psychosocial domain, consists of three main sub-themes:

- The Circle of Women: The Therapeutic Value of Group Exercise highlights the role of shared experiences in overcoming initial fears and self-doubt through being part of a community. The group setting provided emotional support and knowledge exchange, fostering confidence and reducing anxiety. It served as a therapeutic space for discussing emotions and pregnancy experiences, while also encouraging a sense of personal strength and motivation, enhancing adherence to regular exercise.
- Atmosphere: Supportive and Empowering Environment: emphasises the importance of creating an enjoyable space beyond physical activity. The atmosphere encouraged emotional openness, relaxation, comfort, and social connection, built on the shared experience of pregnancy and childbirth. Laughter and free expression contributed to a positive experience, while an empathetic and supportive trainer played a key role in maintaining trust and engagement. The atmosphere was also enhanced through the use of appropriate music and props.
- Social support and influences beyond the programme: this sub-theme explores the impact of external relationships on adherence to regular exercise. Family and partner support encouraged consistent participation, while friendships formed during the programme often extended beyond its duration, positively influencing long-term movement habits and overall well-being.

The second article will address the physical domain, focusing on exercise-related themes such as Structured but Flexible Exercise Programme Design and The Role of the Trainer: Personalised Support and Guidance.

While the third article will explore the psychoeducational domain, with themes including *Reducing Fear and Anxiety Around Pregnancy and Childbirth*, as well as *Psychological Benefits* (see Figure 1).

This three-part approach ensures a comprehensive and nuanced analysis of the factors that contributed to participants’ success in maintaining physical activity during pregnancy, while also addressing the broader dimensions of their transformative experience.

The three domains were organised to provide clarity and structure. However, these aspects are deeply interconnected and cannot be entirely separated from each other. Each domain influences and reinforces the other. This overlap underscores the programme’s comprehensive impact, where no single domain operated in isolation.

### 3.1. “It’s More than Just Exercise”

#### 3.1.1. Programme as a Transformative Experience

This core theme indicates that, for the participants, the programme transcended physical exercise. When talking about the programme, they used terms such as ‘sacred time’, or ‘a natural part of life’—which may indicate their perception of a holistic approach to the programme and its significant value in their lives. Very often, women would describe their participation as an ‘experience’ and would emphasise that for them, it was not ‘just’ about physical exercises, suggesting that in addition to physical activity, there were other important components of the programme that influenced their willingness to participate regularly.

The quotes below highlight that the programme was significant to the women not only for its physical benefits but also for its impact on their psychological and social well-being. Additionally, the programme provided valuable information, making it a multifaceted experience. As a result, it became a transformative journey that fostered personal development and growth, extending far beyond the physical aspects of exercise:

*“In other places [like antenatal educational classes], information is limited, presented in a dry way, and there’s no exercising. But here, everything was included: exercises, breathing, meeting other pregnant women, and a wonderful atmosphere—you didn’t even feel like we were meeting for a fitness class. It felt more like meeting up with friends to laugh, talk, and, additionally, exercise.”*

(Participant 5)

*“The programme was more than just exercise; it created a support system. It gave me friendships that continue to this day and shaped the way I approach my health and well-being.”*

(Participant 10)

*“Participating in the programme was a truly life-enriching experience—very supportive and also strengthening my physical well-being.”*

(Participant 4)

*“Pregnancy was a time of personal growth for me, when (…) I also went to a psychologist for the first time and started learning about my emotions, becoming aware of them. I wasn’t connected to my emotions at all before, and I also touched on this in the programme.”*

(Participant 9)

#### 3.1.2. The Circle of Women—Therapeutic Value of Group Exercise

- Overcoming Initial Fears

Before beginning their participation in the programme, many women expressed having difficult feelings and thoughts around joining. They had concerns about being judged by or compared to others when exercising in a group, especially in terms of physical fitness. This seemed particularly important for women who were inactive before getting pregnant:

*“I was afraid whether I’d manage, whether I could do it (…) for example (…) with coordination, you know, during pregnancy—like everyone else going right, and me going left. I also wondered how I’d be perceived, thoughts about myself, like whether I was good enough, or if I’d come across as clumsy or helpless.”*

(Participant 10)

*“I had some concerns about my fitness levels because before pregnancy, I didn’t lead a particularly active lifestyle and wasn’t the sporty type. I wondered whether I would be able to keep up with the other women.”*

(Participant 3)

*“The beginning was difficult, to be honest—difficult in the sense that I had to catch up with my physical fitness and complete all the exercises. However, by the end of my pregnancy, I felt that I was actually in better shape than when I started.”*

(Participant 5)

- Building Community Through Shared Experiences and Diversity

Initial fears were gradually replaced by a strong sense of belonging as the group evolved into an increasingly supportive community. According to the respondents, this was because the participants on one side were at a similar stage in life but at the same time, at different stages of pregnancy, and who came from various backgrounds and had diverse experiences. The women valued the shared experience of being pregnant, as it created common ground that facilitated bonding and sharing of insights. This helped break down the barriers of comparison and judgement, fostering a sense of commonality and togetherness:

*“I had the opportunity to participate with other women in the same [life] period as me, with the same problems (…) In fact, we exercised, yes, it was in a sense a physical activity, but above all it was very strengthening for me personally.”*

(Participant 8)

*“We also knew what might await us in the next stage [of pregnancy], because we were, as far as the group was concerned, each of us was at a different stage of pregnancy.”*

(Participant 4)

This diversity, combined with shared experiences, fostered a strong sense of equality and mutual respect, where differences were acknowledged but not divisively so. According to the respondents, the lack of hierarchy, including that of the trainer, was crucial in maintaining a supportive environment in the group. Women felt accepted and respected, regardless of their fitness level or pregnancy stage, which encouraged participation and sustained motivation:

*“So well, yes, more of a mental side [was important to me] in a group, because we didn’t compete with each other (…) we were well matched.”*

(Participant 4)

*“[With the trainer] it was a very good, nice collaboration, a nice relationship, without any of that ‘I’m the trainer, and you’re just here to exercise’ kind of attitude. It was a normal, friendly, peer-like relationship, I would say.”*

(Participant 1)

- The Therapeutic Role of the Group

Since all the women were pregnant and navigating similar physical and emotional challenges, the group formed a natural support network. According to the participants, the group eventually became a ‘circle of women’ and a ‘therapeutic group’, where women felt safe to express themselves openly and discuss everything without ‘taboo’. The group acted as a therapeutic space where participants could discuss not only their physical states and pregnancy ailments but also their emotional challenges, fears about childbirth, and concerns about motherhood. As the women said, they became ‘psychologists for each other’, offering advice, sharing stories, and simply being there for one another. Thus, the group became a source of emotional support and psychological relief. For some women, this peer support was instrumental in preventing emotional crises, as noted by Participant 4, who credited the group with helping her avoid an ‘emotional crisis’ during her pregnancy:

*“It was also a kind of, you could say, therapy, because it’s also that kind of support, a circle of other women who are pregnant. And also the realisation that ‘Oh, I’m not (…) alone in this. There’s one, another, and another who are going through the same thing’.”*

(Participant 10)

*“It was a circle of women—supporting each other, learning from each other”*

(Participant 1)

*“We were sort of like psychologists, us for each other, each of us exchanging our experiences (…)”*

(Participant 4)

The participants observed that the desire to meet with other pregnant women and the relationships formed between the group members at a deeper level of emotional engagement contributed to creating a sense of belonging and a holistic support system that encouraged regular attendance.

- Peer Motivation

The participants also spoke about their initial difficulties in finding and sustaining motivation to exercise on their own, particularly when dealing with the physical and emotional demands of pregnancy, which they described as a very specific period in their lives, different from other periods. Participants described pregnancy as a unique ‘state’, distinct from everyday life, marked by both easier and more challenging days, including emotional and physical crises. Some women experienced severe fatigue and nausea in the first trimester, making daily functioning and work difficult, while others found the third trimester most challenging due to exhaustion and limited mobility. Emotional fluctuations, such as heightened sensitivity and intense mood swings, were also common, although participants found these changes harder to accept and perceived them as less desirable.

However, a difference between the participants was observed. Those for whom some form of physical activity was already a part of their lives before pregnancy (see Table 1) spoke about their internal conviction and motivation to continue exercising during pregnancy. It seems that they already had an internalised motivation to remain so. These participants spoke of a strong determination to exercise during pregnancy, describing the enjoyment they derived from physical activity, saying they liked exercising, and were also aware and convinced of the benefits of physical activity:

*“It was my internal need [to be physically active].”*

(Participant 8)

*“I just do it on my own because I know from my own experience that physical activity really has a positive effect on overall well-being.”*

(Participant 4)

Conversely, participants whose lifestyles before pregnancy were more sedentary seemed to have more difficulty starting physical activity during pregnancy and needed external support to build this motivation. The participants mentioned that they initially found it difficult to motivate themselves to exercise. Some were convinced and encouraged to participate in the programme by their husbands or partners, while others were motivated by friends or acquaintances. The women said it was easier to decide to participate in the programme together with a friend:

*“I decided to participate partly because my husband encouraged me, saying, ’You have to do something during these 9 months’.”*

(Participant 4)

*“At the time, my husband said, ‘Go, go, go, go, go.’ Basically, he was pushing me to go, not in a way of motivating me by saying it would be better for me, but more like, ‘If you want to go, then go, so later you don’t say it’s my fault you didn’t go’”*

(Participant 2)

*“It was actually my friend who also told me, ‘Go, I assure you it will make things easier’. She was really convincing me to give birth naturally and kept saying, ‘You absolutely have to go to participate in this programme’.”*

(Participant 2)

Participants repeatedly emphasised that exercising in a group was more motivating, helpful, and enjoyable than exercising alone at home or at a gym. Some participants pointed out that exercising in the gym focuses more on the purely physical aspect, and concluded that this was not their preferred form of physical activity. This also confirms that the participants needed other sources of support to maintain their commitment to fully engage in the exercise programme:

*“It was important to me that these were group classes. I never liked the gym as just strength training exercises.”*

(Participant 4)

*“Well, like I said, I wouldn’t go to the gym alone because I don’t have enough knowledge about exercises, and I would be afraid that I might harm myself—or even more so, that I could harm my baby.”*

(Participant 6)

*“[The classes were a] friendly place, the opposite of the gym. I’ve never liked the gym or strength training on its own. I preferred group activities, especially in women’s clubs—that’s where I felt best.”*

(Participant 4)

The presence of other pregnant women helped create a motivating environment. Seeing others engaged in the same activities encouraged participants to push through moments of low motivation or physical difficulties. The women noted that, during the programme, there were days and moments when they faced challenges, reluctance, or lacked the energy to exercise. According to them, the group was very important because the participants could draw motivation from each other. Exercising in a group provided a collective form of motivation that many women would struggle to achieve on their own, which they described as ‘contagious’. Knowing that others in the group were facing similar difficulties but still showing up created a positive pressure to keep attending, helping them stay committed. The phrase ‘catching each other’s motivation’ which repeatedly appeared in the interviews, reflects the contagious enthusiasm, energy, and resilience fostered within the group:

*“During the classes, I drew motivation from the other women exercising.”*

(Participant 2)

*“I would leave those classes so energised. Not only because of the endorphins after exercising, but also because of the other women there, all with their bumps just like mine. They were working just as hard, always with a smile on their faces. That was amazing; it was very important and motivating for me.”*

(Participant 8)

*“First of all, definitely the group—that being in a group gave motivation. When one person went, we all went again, even if sometimes you didn’t feel like it, like when you were tired. Toward the end, I didn’t always feel like going, but I thought, ‘No, I’m going’ because it’s something to get out for. And once I went and exercised, everything felt completely different afterward.”*

(Participant 1)

*“Sometimes I wanted to just lie down, but I felt that I had to go, like the others do, so I went.”*

(Participant 7)

The appropriate size of the group was also highlighted as a key factor, as it allowed for meaningful connections both among participants and with the trainer, facilitating a more personal and supportive atmosphere.

*“Our group—there weren’t many of us; we had quite an intimate group, and that was nice too. Yes. There were maybe 6 or 5 of us, I don’t remember exactly, but it wasn’t a big group. It was different from being in a large group with many women; it was smaller, and that meant there was time for us and more contact with the trainer.”*

(Participant 10)

*“The fact that our group was small made a difference—it was a nice, cosy group. (…) It wouldn’t be the same if the group were too large, with too many women. A smaller group means you’re not anonymous, and the trainer is in close contact with us.”*

(Participant 7)

*“Yes, when a few women come to the sessions, and they’re going through similar experiences, it just makes it more cheerful and interesting.”*

(Participant 1)

#### 3.1.3. Atmosphere: Supportive and Empowering Environment

- Emotional Climate: Enjoyment and Comfort

The interviewees strongly emphasised the importance of the atmosphere, describing it as highly positive and pleasant. They portrayed the atmosphere as a blend of collective emotional and social tones experienced by the group. This atmosphere was shaped by the mood, interactions, and emotions that participants subjectively encountered in the group setting:

*“[The most important thing was] the atmosphere, mainly the atmosphere, because I would get up and go, I just liked it. And then, over time, as my belly grew, I could see that I felt good, and those were the two main driving forces for me: the atmosphere and the fact that I felt really good after those classes.”*

(Participant 4)

*“I just wanted to come to those classes. Sometimes, of course, you’d rather stay at home, maybe skip it—’I’m not going…’ because the classes were only two or three times a week. So sometimes I’d think, ‘No, I won’t go tomorrow…’ but then I’d feel like—‘Today, I have to’.”*

(Participant 8)

Enjoyment emerged as a crucial aspect of the participants’ experience, with many women emphasising the pleasure they derived from attending the classes. This sentiment is reflected in the frequently repeated phrase, “*It was just a pleasure to come to these classes*”. Participants consistently highlighted the presence of comfort and enjoyment during the sessions. These elements contributed to a positive and engaging atmosphere that encouraged regular attendance and sustained participation:

*“Very very pleasant(…) it was that kind of setting, other women—always smiling. The trainer—also always so positive. We jumped around, jumped around—figuratively speaking, of course. But overall, very, very positive.”*

(Participant 8)

*“The pleasant atmosphere was important. It was motivating, and you could say also informative, because the meetings weren’t just about exercising but also about sharing stories and giving each other advice.”*

(Participant 2)

- Freedom to Express Emotions

The participants also emphasised the emotional dimensions of the atmosphere, noting their appreciation for the light-hearted interactions among group members. They spoke about ‘having good fun’ and shared that ‘it was important that there was laughter’. This highlights the significance of being able to express their emotions freely during the sessions, without the fear of judgement, which contributed to their positive experience:

*“It was cheerful. It wasn’t like, you know, you weren’t allowed to smile. It was genuinely, you could say, all in a pleasant atmosphere.”*

(Participant 2)

*“There were jokes in between. Nice, really nice… nice, nice, really nice.”*

(Participant 7)

*“There were laughs, and good times—it was more than just exercise.”*

(Participant 1)

The camaraderie and the presence of joy, laughter, and lightness in the form of jokes and conversations fostered an emotional uplift that enhanced the participants’ attendance. The women were not merely exercising for the sake of physical fitness. They were engaging in an activity that provided emotional enrichment in a supportive and uplifting atmosphere. This made the exercise experience more holistic and personally rewarding, as it combined physical activity with emotional and social fulfilment:

- The Trainer’s Role in Fostering a Supportive Atmosphere

The trainer played an essential role in fostering this atmosphere. Many women spoke about the trainer’s positive attitude, frequently mentioning that her approach to facilitating the classes, as they remembered, ‘always with a smile on her face’ helped them feel positive and motivated to continue exercising. Her attention to each participant’s individual needs, supportive approach, and ability to build trust were all crucial.

Additionally, the participants spoke about an atmosphere full of engagement, presence, and attentiveness—from the trainer—where ‘time passed quickly’ (Participant 3).

Moreover, the trainer’s ability to unite the group fostered a sense of togetherness and mutual support. Participants highlighted her dual role as a professional and a source of emotional support. This supportive relationship was fundamental in creating an atmosphere in which the participants felt connected and empowered.

Through her expertise, energy, and empathy, the trainer became a central figure in shaping the group’s experience, ensuring that the classes were not only physically beneficial but also emotionally uplifting and unifying:

*“And she [the trainer] would say, ‘Now we’re doing this, now we’re doing that,’ and somehow everything was very, very positive. I think Beata was the most positive figure for me; thanks to her, I didn’t get discouraged after the first or second session, thinking I couldn’t manage. Instead, I always came back with a smile and positive energy because of her.”*

(Participant 6)

*“I think the atmosphere during these classes was all about shared motivation, collective experience, and preparing for this period. That was probably the most important thing, and it would have been missing if our trainer hadn’t been so contagious with her positive energy. I believe that, over time, the exercises alone would have become boring without it.”*

(Participant 5)

*“She would calm us down, explain everything, and that’s what made us feel safe.”*

(Participant 7)

*“It was also largely thanks to Beata because she knew how to bring everyone together. Of course, she’s a professional and focuses on what she does, but at the same time, she was a source of support for us.”*

(Participant 4)

- Physical and Structural Elements

Physical and structural elements also played important roles in fostering a supportive and engaging atmosphere during the exercise sessions. A well-designed space—clean, well-lit, and comfortable—created an inviting environment that also encouraged participation. Music, whether energising or calming, set the tone for the session and enhanced motivation.

*“The music always ‘disarmed’ us, it energised us to start the workout with a smile on our faces.”*

(Participant 7)

*“Beata would also play us relaxing music (…) It gave us a moment to just be with ourselves and also with our little ones. It was like the finishing touch to the sessions. By then, we were a bit tired from the exercises—it wasn’t always easy—but when you lay there on the mat or the floor, listening to the music, and she’d say, ‘Place your hands on your belly,’ it was such a lovely experience.”*

(Participant 10)

Additionally, a varied, engaging, and well-paced session structure and the use of various props ensured that participants remained focused and satisfied, contributing to a positive and enjoyable experience. Together, these elements establish an atmosphere that promotes both physical activity and emotional well-being:

*“Every exercise session was truly different. Each meeting, even though Beata had her planned repertoire of exercises, was never repetitive. The exercises were varied. We had sessions with exercise balls, sessions at the barre—some of them looked like ballet exercises. We had fun warm-ups inspired by aerobics, exercises with dumbbells and resistance bands, and general conditioning workouts, working the whole body from head to toe.”*

(Participant 3)

*“The exercises were very engaging, and various equipment was used, such as balls, resistance bands, and bars, which prevented monotony.”*

(Participant 9)

*“The sessions were diverse, never repeating, and included both large and small equipment.”*

(Participant 3)

- Shared Purpose

A shared purpose was also an influential element in fostering the right atmosphere within the group during the exercise sessions. In this context, the common goal of preparing for childbirth created a strong sense of unity and connection among the participants. This shared focus not only reinforced their commitment to attending sessions but also cultivated a supportive community in which women felt understood and motivated.

The collective experience of preparing for childbirth allowed participants to relate to each other’s challenges, fears, and aspirations, transforming the group into more than just an exercise class. The shared purpose also gave the sessions deeper meaning, as the women were not only working on their physical health but also building confidence and resilience for the life-changing event of giving birth. This alignment of goals created a collaborative and emotionally uplifting environment, strengthening group cohesion and enhancing the overall experience:

*“I think it was the atmosphere during those classes—the shared motivation, the shared experience, preparing for this period and birth—that was the most important.”*

(Participant 8)

*“It strengthened me to see that, you know, everyone had similar problems to some extent, but they always left with a smile and managed to handle everything, especially childbirth.”*

(Participant 6)

*“[The atmosphere] was very positive because it was women who also, you know, sometimes had back pain, or their own pregnancy-related discomforts. But we were going through it together, experiencing it together, preparing for birth together (…) we supported each other(…). It was very, very pleasant.”*

(Participant 10)

#### 3.1.4. Social Support and Influences Beyond the Programme

- Social Support as an Enabler

Family support also emerged as an important factor in enabling and sustaining physical activity during pregnancy. The participants emphasised that the encouragement and assistance they received from their families, particularly their partners and family members, played a significant role in their ability to attend regular sessions. This support often manifested in practical ways, such as help with household responsibilities or caregiving, and emotional reinforcement of their commitment to staying active. For many participants, the approval and encouragement of family members provided the necessary motivation to overcome their initial hesitations about engaging in physical activity during pregnancy. As one participant noted, her husband’s consistent reassurance helped her prioritise her well-being and view exercise as an essential part of her routine:

*“The support of my partner and family was crucial, for example, knowing that I could sometimes leave my daughter with her grandmother. Family support was a significant factor that made it possible for me to take part in this experience.”*

(Participant 8)

*“My family was very happy with what I was doing and supported me fully. They did everything they could so that I could attend the sessions, right up until the birth—every week or twice a week, depending on how the sessions were scheduled.”*

(Participant 7)

- Enduring Social Connections and Sustainable Impact

The connections and friendships formed within the group extended beyond the duration of the programme, impacting the women’s lives in meaningful ways. Many women reported maintaining friendships with fellow participants long after the programme had ended, valuing these connections as an enduring source of support in navigating motherhood. The programme thus provided not only a space for physical activity but also a foundation for meaningful social bonds that enriched their lives during and beyond pregnancy.

This highlights the value of the programme’s social dimension, as it provided a space for lasting relationships that continued to offer support even after the structured sessions had ended:

*“I’m still in touch with the girls; I made friendships that I still maintain, and our kids still play together.”*

(Participant 6)

*“I also opened up a bit; those classes with other women, new acquaintances, new friends, then some later get-togethers… Yes, it was all very, very positive and uplifting.”*

(Participant 8)

*“During the programme, I became friends with another mum who had a daughter born a little earlier, and it turned out that our girls later ended up in the same preschool group. So, in a way, we also shared this experience—first, we exercised together, and now our daughters are ‘exercising’ together too.”*

(Participant 9)

Participants reported lasting lifestyle changes following their involvement in the “Conscious 9 Months” programme, with many incorporating more movement and exercise into their daily routines even after the programme had ended. They described gaining a deeper appreciation for physical activity, which motivated them to maintain regular exercise as part of their postpartum lives. Some participants also emphasised that their experiences influenced their approach to parenting, as they became more conscious of promoting a healthy lifestyle for their children. They actively encouraged physical activity within their families, ensuring their children understood the importance of movement and well-being from an early age:

*“Before pregnancy, I wasn’t as aware and active as I became afterward. Now I know how important physical activity is not only for me but also for my child. After these classes, I started to think differently about movement and how it affects both the body and the mind.”*

(Participant 7)

*“I was always physically active to some extent, but not at this level. Now, having children, I understand that I need to set an example and show them that movement and good nutrition are essential. This program helped me integrate this awareness into my everyday life.”*

(Participant 6)

*“It was very important to develop body awareness, not just during movement but also in relaxation—to be able to calm down and unwind. And looking back now, I believe that body awareness is key. Since my participation in the programme, I keep deepening it to this day.”*

(Participant 9)

Participants expressed a sense of loss and nostalgia after the programme concluded, underscoring the profound impact the group had on their lives. The group did not just provide temporary support. It became a significant part of their pregnancy journey, which they continued to cherish:

*“There was a great atmosphere. The women would come, we’d laugh together, and it was such a warm environment. Yes, I miss that.”*

(Participant 5)

*“Those were amazing classes back then. I felt great, and during my second pregnancy, I couldn’t participate in any classes, and I missed it so much. In my second pregnancy, I had to stay in bed until the end of the third month, and afterward, I had to be very cautious, and I really missed it.”*

(Participant 10)

*“I wish those classes were available all the time because, for example, during my second pregnancy, they weren’t, and it just wasn’t the same.”*

(Participant 3)

## 4. Discussion

During pregnancy women seek support through meeting with healthcare professionals, discussing experiences with friends and family, and seeking additional learning opportunities. Education and advocacy are two of the most important aspects of being a healthcare professional specialising in women’s health [65].

This qualitative study explored the experiences of participants involved in the “Conscious 9 Months” programme, designed as a holistic prenatal exercise intervention, integrating physical activity, social interaction, emotional support, and education to comprehensively address the needs of pregnant women (see Figure 1). Grounded in existing research [37,66], the physical dimension of the programme focused on individually tailored exercises, ensuring safety and effectiveness under the guidance of an experienced prenatal exercise trainer (see published protocol for details [7,57]). Group-based sessions were designed to enhance peer support and motivation, as social connections are known to improve adherence and engagement [38,67]. The programme also fostered emotional expression and a supportive atmosphere, promoting psychological well-being and group cohesion [68]. Educational elements were embedded throughout the programme, covering the safety of prenatal exercise, childbirth preparation, and postpartum physical activity [29,68,69]. Crucially, the trainer played a dual role, combining professional expertise with a highly supportive approach, which was instrumental in fostering trust, motivation, and a sense of security among the participants.

In this article, the psychosocial dimensions, including the appropriate atmosphere, are discussed in more detail.

### 4.1. Holistic Approaches in Prenatal Care

A holistic approach to health considers multiple dimensions of well-being, including physical, mental, emotional, social, intellectual, and spiritual factors. In the context of pregnancy, psychological wellness is particularly significant, as it can influence pregnancy outcomes [70]. Holistic prenatal care has been shown to reduce stress and anxiety, enhance emotional resilience, and improve overall well-being, ultimately leading to better outcomes for both the mother and child [68]. Importantly, a prenatal holistic lifestyle approach empowers women to engage in regular physical activity. As noted by Newson et al. [49], pregnant women continuously navigate personal beliefs, the health of their unborn babies, and social influences while seeking information to support healthy behaviours. This is why addressing these different dimensions via holistic programmes, is crucial.

Our findings revealed that the success of the “Conscious 9 Months” programme in supporting women during pregnancy was not solely attributable to its physical exercise component. The present study also found that participants valued the comprehensive nature of the prenatal exercise programme, which addressed not only physical health, but also emotional well-being, and social support. This multidimensional framework effectively addressed a variety of needs simultaneously, making the programme more appealing and impactful for participants, and enabling regular attendance at sessions.

### 4.2. Psychosocial Dimensions of the Programme

#### 4.2.1. Group Versus Individual Approach

Physical activity during pregnancy is commonly undertaken in two contexts: group-based settings (e.g., structured classes) or individually-based settings (e.g., home-based) [71]. A growing body of literature highlights the benefits of a group-based approach. For instance, Dishman and Buckworth [72] synthesised results from 127 studies involving approximately 131,000 participants who took part in physical activity interventions across community, school, workplace, home and healthcare settings. Their analysis revealed that group-based interventions produced significantly greater effects compared to interventions delivered in other formats (e.g., individually, to families, or to individuals within a group). Similarly, a review by de Castro et al. [25] found that exercise participants report higher motivation in a group setting.

In this study, group exercise sessions provided a distinct experience compared to exercising alone. Participants emphasised the social dimension as a key factor in maintaining regular attendance. This is in line with the study by Livingston et al. [50], who emphasised that pregnant women actively seek “like-minded” individuals to share experiences and gain motivation for physical activity. Given the unique nature of pregnancy, the participants in their study felt that only other pregnant women could truly understand their experiences. In the absence of structured prenatal programmes, many turn to online groups to fulfil their need for community [73]. However, as their pregnancies progressed, the participants reported that face-to-face interactions were significantly more effective in fostering meaningful relationships and emotional support [50]. Our findings align with this, as participants highlighted that the group-based exercise setting cultivated a sense of belonging. The shared experiences and mutual encouragement within the group were instrumental in enhancing adherence to exercise.

#### 4.2.2. Group Exercise and Group Therapy

Participants described the group as a ‘circle of women’ and a ‘therapeutic group’. The holistic group exercise programme for pregnant women shares several notable similarities with Yalom’s general principles of group therapy. Yalom [74] identifies key therapeutic factors such as universality, group cohesiveness, and altruism, all of which were evident in the experiences of women participating in the “Conscious 9 Months” programme. During the sessions, there were moments when they could openly share their emotions and challenges without the fear of judgement. This aligns with the therapeutic principle of universality, as women found comfort in realising that their experiences and struggles were shared by others.

Group cohesiveness, a cornerstone of Yalom’s framework, was reflected in the emotional bonds and mutual support cultivated among the participants. The supportive and non-competitive environment allowed the participants to feel accepted and understood, fostering a sense of belonging. Moreover, the participants frequently acted as sources of support and encouragement for one another, embodying Yalom’s concept of altruism as they shared advice, experiences, and reassurance that helped others navigate their pregnancies with confidence.

The emotional climate of trust and openness in the exercise classes also created opportunities for emotional expression, which Yalom views as crucial for therapeutic outcomes. This climate not only supported participants’ physical activity but also addressed their psychological and emotional needs, creating a holistic environment that resembled the multidimensional benefits of group therapy. Both the exercise programme and group therapy underscore the profound impact of shared experiences and social connections in promoting well-being, which was evident when the participants repeatedly mentioned that they just wanted to come to the classes.

Research comparing group exercise programmes and group therapy highlights both the shared and distinct benefits of these interventions [75,76]. Both approaches emphasise the value of social support and emotional connection, which are central to their effectiveness. Group exercise programmes often provide participants with a sense of community and motivation through shared physical activities, fostering emotional well-being and reducing feelings of isolation, which is especially important during pregnancy. Similarly, group therapy leverages interpersonal dynamics to promote self-reflection, emotional expression, and personal growth.

Studies have shown that group exercise can yield psychological benefits similar to those of group therapy. For example, physical activity in groups has been found to significantly reduce anxiety and depression, often paralleling the outcomes of structured cognitive behavioural therapy (CBT) sessions. In randomised trials comparing group CBT with aerobic exercise groups, both demonstrated improvements in depression and anxiety, with exercise contributing additional physical health benefits [75,76].

The emotional climate of group settings—whether for exercise or therapy—is a key mechanism of change. Group cohesion, mutual encouragement, and opportunities for emotional expression were common across both modalities. However, while group therapy often focuses explicitly on psychological processes like cognitive restructuring, group exercise programmes integrate physical activity as a pathway to both mental and physical health, making them appealing for prenatal holistic interventions.

#### 4.2.3. Enhancing Motivation: Key Differences Between Active and Inactive Women During Pregnancy

Kahn et al. [77] and Carron et al. [78] found strong evidence supporting the effectiveness of physical activity interventions that incorporate social support. Their studies demonstrated that involvement from others—such as members of cohesive exercise classes, social collectives, supportive family and friends, or engaged professionals and researchers—can significantly influence both participation in physical activity and the benefits derived from it. Exercising in a group setting has been shown to increase participants’ motivation, which is crucial for maintaining exercise regularity [25,79].

Group-based prenatal exercise programmes play a crucial role in enhancing motivation for physical activity among pregnant women. Studies have indicated that the presence of a supportive social environment fosters a sense of accountability and shared commitment, making women more likely to maintain regular participation [38,67]. The shared experiences within a group setting not only provide emotional reinforcement but also alleviate stress and anxiety, which can be significant barriers to physical activity during pregnancy [29,80]. Collado-Mateo et al. [81] further highlighted that pregnant women who exercise in a group setting report greater enjoyment and psychological well-being, making them more likely to remain active.

Similarly, the findings of this study highlight that the “circle of women” created a unique and invaluable combination of physical activity with social and emotional reinforcement. The opportunity for social interaction and meaningful conversations emerged as key motivators for participants to consistently attend classes. Within the group, motivation was described as contagious—particularly from women who appeared more motivated to those with less motivation—as members inspired and encouraged one another to remain engaged in the programme and provided support during more challenging moments. Thus, the presence of other women and the possibility of mutual support had a positive impact on the participants’ commitment to regular exercise.

Other studies have also shown that the motivational impact of group-based programmes is particularly important for women who were previously inactive, as they benefit from peer encouragement and positive social influence [68]. As Hegaard et al. [27] state, most women who were physically active before pregnancy continue exercising during pregnancy due to their well-established habits and strong motivation. However, pregnancy-related physical changes and discomfort often lead to a decline in activity levels, even among women who were previously active. This decline can be mitigated by expert guidance from prenatal professionals and support from pregnant women. The current study supports this, demonstrating that when both elements—a qualified prenatal trainer and a supportive exercise group—are present, women are significantly more likely to maintain regular physical activity despite pregnancy-related difficulties.

Interestingly, Newson et al. [49] reported that even women who were physically active before pregnancy often stopped exercising due to concerns about safety. A key finding from their study suggests that access to prenatal lifestyle interventions providing reassurance and guidance on safe exercise practices encourages women to remain physically active.

Findley et al. [51] also discuss the distinction between women who were physically active before pregnancy and those who were inactive, demonstrating that previously active women were more likely to continue exercising during pregnancy. This suggests that special attention should be given to women who begin exercising during pregnancy, as they may require additional support from both the trainer and the group to maintain participation.

Furthermore, many women experience social pressure to conform to the prevailing attitudes toward physical activity during pregnancy [51]. Their perceptions are shaped by comparisons with others and by hearing accounts of women who either maintained or discontinued exercising. In the current study, the participant group included both previously active and inactive women, highlighting the crucial role of peer influence. Those who had already developed an intrinsic motivation to exercise could serve as role models, indirectly shaping the attitudes of those who required greater support in building their motivation.

#### 4.2.4. Enhanced Adherence Through Group-Based Exercise

Sustaining adherence to exercise programmes during pregnancy is significantly influenced by the social dynamics and structure of group-based sessions. Research suggests that women who engage in structured group activities demonstrate higher levels of commitment compared to those who attempt to exercise alone [25,72]. This was confirmed by the participants in our study, who stated that they would probably not have exercised alone at home. The opportunity to build connections with other pregnant women fosters a sense of belonging that enhances long-term engagement [38].

In the current study, the participants’ group experiences while performing exercise played a crucial role in enhancing adherence to physical activity. Participants repeatedly highlighted that being part of a group fostered a sense of accountability and collective motivation, reinforcing the findings of Dishman and Buckworth [72] and Carron et al. [78], who demonstrated that social engagement in exercise settings enhances commitment and consistency. The above observations from our participants align with the existing literature. Regarding the effect of social influence on exercise adherence, Dishman and Buckworth [72] and Carron, et al. [78] emphasised that exercising in a group setting leads to greater adherence compared to exercising alone.

The supervision of programmes by qualified exercise professionals has been shown to enhance adherence by ensuring participant safety [82]. Positive social interactions with both staff and other participants have also been identified as key facilitators of adherence. Similar to the findings of the current study, Livingston et al. [50] also highlighted the critical role of a prenatal exercise trainer in facilitating continued physical activity. Personalised guidance and an individualised approach provided by a knowledgeable professional were found to be decisive factors in supporting women in exercising consistently throughout pregnancy.

At the same time, limited adherence can be attributed, in part, to poor follow-up of control groups compared to intervention groups or to pregnancy-related adverse events leading to dropouts [82].

### 4.3. The Role of the Atmosphere

As revealed in our study, the atmosphere was a crucial factor in fostering regular attendance. Feelings of joy, fun, comfort, and relaxation were essential aspects of the sessions for participants. Informal expressions of emotions and nonverbal elements played pivotal roles in fostering group dynamics and enhancing engagement.

Shared jokes and light conversations, laughter, and the free exchange of thoughts gave the sessions a pleasant “feel”, which was repeatedly brought up by the interviewees. The co-created environment demonstrated that the social and emotional elements of prenatal exercise programmes are as crucial as their physical aspects.

Enjoyment serves as an immediate reward that may increase adherence more effectively than long-term health benefits do. Conversely, unpleasant experiences can reduce adherence, a phenomenon commonly observed in patients with musculoskeletal pain [81].

This issue was particularly highlighted by our participants, who expressed great enjoyment of the exercise sessions, describing them as a source of pleasure.

Creating a positive atmosphere during prenatal exercise classes is crucial for enhancing adherence, as it fosters a sense of comfort, emotional expression and enjoyment. Other studies have also shown that pregnant women highly value an environment where they feel at ease, can freely express their emotions, and engage in informal conversations with peers, as these aspects reduce stress and enhance their overall well-being [68,80,83]. Group-based prenatal exercise programmes that incorporate elements of laughter, joking, and social interaction have been found to increase motivation and commitment, as they transform exercise into a pleasurable and socially enriching experience [25].

The role of the trainer in co-creating this atmosphere is particularly significant, which was often emphasised by the participants in this study. A knowledgeable and empathetic instructor not only provides professional guidance but also cultivates a supportive and engaging environment that encourages continued participation [81]. Trainers who establish a non-judgemental, open setting, where women feel empowered to share their concerns and experiences, contribute to a sense of belonging and trust, which further reinforces adherence [50,81]. Moreover, small, well-organised groups led by experienced instructors have been found to yield the best results in terms of engagement and retention, as they provide individualised attention while fostering peer support [51].

Furthermore, research suggests that integrating relaxation elements and a flexible, participant-centred approach enhances adherence by accommodating the physical and emotional needs of pregnant women [68,83]. When sessions incorporate structured but adaptable activities, humour, and social interaction, women are more likely to feel motivated and committed to maintaining regular exercise throughout pregnancy [50].

Women participating in our programme also highlighted the importance of variety and attractiveness in the sessions, particularly the use of diverse exercises and equipment, such as balls and bars, as key factors in avoiding monotony. As one participant summarised, “Otherwise, it would be boring” (Participant 8). Some authors have confirmed this, linking high adherence rates (>80%) to the inclusion of varied exercises [84]. While variety was appreciated, the inclusion of consistent and repeated elements provided a reassuring structure, which was equally valued.

The structured yet flexible nature of the programme, combined with the presence of a supportive social environment, allowed women to feel both physically challenged and emotionally secure. Thus, the ability to create a comfortable environment and form homogeneous groups plays a significant role in sustaining engagement in exercise [81]. In summary, ensuring that prenatal exercise classes prioritise a positive, emotionally supportive, and enjoyable atmosphere—facilitated by both the group and the instructor—can significantly influence women’s willingness to engage in and sustain physical activity.

### 4.4. Extending Beyond the Programme: Social Support

The results of the review by Al-Mutawtah et al. [34] indicate that pregnant women experienced and valued a wide range of emotional support from various sources, including their female networks, partners, families, and parents.

Qualitative evidence from the study by McLeish and Redshaw [85] suggests that peer support can help reduce low mood and anxiety by overcoming feelings of isolation, helplessness, and stress, supporting improved self-esteem, self-efficacy, and parenting competence. The identified benefits for maternal mental health and well-being indicate that peer support is a promising and valued intervention at a critical time during the transition to parenthood.

Women’s emotions are regulated through social support and relationships, which, in turn, may reduce emotional exhaustion during pregnancy due to fear of childbirth. Family and partner support were highlighted as crucial external factors that facilitated regular participation by the participants in our study. Women appreciated their partners and families endorsement and encouragement of their involvement, reflecting the broader role that social support systems play in sustaining engagement.

Social support is particularly significant, as highlighted by Newson et al. [49], because women often rely on advice from family and friends when making decisions regarding exercise. If those around them discourage physical activity due to concerns about potential harm, women are more likely to reduce or cease exercising altogether. This is consistent with the findings of Findley et al. [51], where participants reported confusion when their close social circle advised them to stop exercising despite professional recommendations encouraging physical activity. In this study, encouragement and support from close family members and friends played a crucial role in enabling the participants to sustain their attendance.

The continuation of social relationships and the expressed sense of loss after the programme ended indicate that the support network established was deeply meaningful. They wished they could continue their participation after finishing since, as they emphasised, the group was not just temporary support. It became a significant part of their pregnancy journey. This suggests the need for ongoing support mechanisms beyond the structured programme to maintain these beneficial connections. Participants strongly emphasised that their involvement in the programme not only supported them during pregnancy but also facilitated long-term lifestyle changes. Many described how the programme helped them establish and maintain regular physical activity habits, which they continued postpartum. Additionally, they highlighted how their experiences influenced their approach to health and well-being, extending beyond their own routines to shaping their families’ behaviours. Several participants reported that they actively promoted physical activity among their children, incorporated movement into their daily lives, and fostered a positive attitude toward exercise from an early age. This aligns with previous research demonstrating that maternal engagement in physical activity during pregnancy can have a lasting impact on family health behaviours. In the study by Livingston et al. [50], it is shown that the friendships and social ties formed in these programmes often extend beyond pregnancy, reinforcing lasting healthy behaviours and sustained physical activity. These findings underscore the transformative potential of group-based prenatal exercise programmes, not only in enhancing short-term maternal well-being but also in fostering sustainable, intergenerational health benefits.

### 4.5. Strengths and Limitations

This study has several strengths, particularly its in-depth exploration of pregnant women’s lived experiences with group-based physical activity. The qualitative approach provided a rich and nuanced understanding of the psychological and social benefits of participation, which quantitative research may overlook [28]. A key strength was also the ability to assess the long-term impact of the programme, as interviews were conducted five years after participation, allowing for insights into sustained behavioural changes, social connections, and lifestyle modifications. Additionally, this study highlights the role of social support in exercise adherence, aligning with research demonstrating that group-based physical activity fosters motivation, emotional resilience, and a sense of community [25]. Efforts to minimise bias were also undertaken, including conducting interviews with a neutral psychologist rather than the first author, who had served as the participants’ trainer.

However, some limitations of this study should be acknowledged. The time gap between programme participation and interviews may introduce recall bias, as retrospective accounts may be influenced by selective memory or idealisation [62]. Additionally, self-selection bias is a potential concern, as participants who successfully adhered to the programme were more likely to participate in the study, meaning perspectives from those who dropped out or chose not to engage in prenatal exercise were underrepresented [64]. The study’s findings may also have limited generalisability due to the homogeneity of the sample, as all participants were from the same cultural and geographical context. Finally, while this study demonstrates the benefits of group exercise, it does not directly compare group-based and individual exercise experiences, which could provide further insight into the role of social support in adherence. Despite these limitations, this study offers valuable contributions to understanding how social and emotional factors influence engagement in prenatal physical activity and provides practical implications for designing more effective exercise interventions for pregnant women.

### 4.6. Implications for Practice

Group exercise programmes, particularly those with holistic designs, may offer therapeutic benefits while also addressing physical health goals.

Future prenatal programmes can benefit from adopting holistic frameworks that encompass different aspects, including physical, social, emotional, and educational. Such approaches are better equipped to meet the comprehensive needs of pregnant women, thereby fostering greater adherence. When designing such prenatal programmes, the following suggestions for conducting them should be considered:

1. Building a Supportive Social Environment
  - Small Group Size: The study highlights that an optimal group size of 6–8 participants fosters a sense of safety, enabling individualised attention from the instructor while facilitating peer support.
  - Prenatal Classes as a Safe Therapeutic Space: Group sessions should foster a supportive environment in which participants feel comfortable sharing their concerns and experiences. This can be achieved by ensuring that the qualities of universality, group cohesiveness, and altruism are present. Creating opportunities for emotional expression, social support, and emotional connection is of utmost importance. This is enhanced when the group consists of pregnant women at different stages of pregnancy and with different life experiences.
  - Utilising Group Motivation: Women are more likely to remain engaged in a structured group setting. Ideally, the group should include both women who were physically active before pregnancy and those who were not, as peer influence is a strong motivating factor.
2. Creating a Supportive Atmosphere
  - Comfortable and Intimate Environment: The training space should be quiet, private, and free from distractions to foster concentration and relaxation. Large gym settings may feel impersonal and discourage participation.
  - Encouraging Emotional Expression: It is essential to foster a positive and emotionally supportive atmosphere. Opportunities for laughter, informal interactions, and open emotional expression should be actively encouraged as they contribute to a sense of community and enjoyment. Both group dynamics and the instructor play crucial roles in shaping this atmosphere, ensuring that participants feel comfortable and supported. Prioritising these elements in prenatal exercise classes can significantly influence women’s willingness to engage in and sustain physical activity throughout pregnancy. A knowledgeable, empathetic, and supportive instructor plays a crucial role in creating an appropriate atmosphere.
  - Essential Equipment: The programme should provide mats, exercise balls, rollers, blankets, cushions, and resistance bands to ensure comfort and versatility in movement.
  - Appropriate Music Selection: Music should align with different phases of the session, ranging from more dynamic beats for the warm-up, moderate tempo during strength exercises, and calming sounds during relaxation.
3. Supporting Long-Term Health and Well-Being
  - Encouraging Partner and Family Involvement: Programmes should integrate strategies that actively involve partners and family members (e.g., prenatal workshops or information sessions) to reinforce long-term engagement.
  - Sustained Social Connections Beyond the Programme: The study found that women continued their friendships after the programme ended, emphasising the importance of follow-up initiatives such as alumni groups, online forums, or informal meet-ups.
  - Sustaining Engagement Beyond Pregnancy: Ensuring continuity of support through postnatal movement programmes, mother-baby exercise sessions, and postpartum recovery groups can promote long-term adherence to movement practices.
  - Expanding access to specifically designed holistic prenatal exercise programmes is crucial, as many women report a lack of suitable opportunities that integrate physical activity with social and emotional support. Developing and promoting such programmes can help ensure that more pregnant women have access to structured, supportive environments that encourage regular participation and overall well-being.

### 4.7. Future Research

While this study provides valuable insights into the psychological, social, and long-term benefits of group-based prenatal exercise, several areas warrant further exploration.

Firstly, future studies should expand the diversity of participant samples, including women from various geographical locations and cultural, ethnic, and socioeconomic backgrounds. Existing research has shown that access to prenatal exercise programmes, cultural perceptions of physical activity, and structural barriers can significantly differ across populations [82]. Such studies would also enhance the representativeness of the findings, providing a more comprehensive understanding of how these factors may influence adherence and long-term health behaviours, and could help tailor interventions to better meet the needs of each diverse group.

Future research should also investigate longitudinal outcomes to assess how participation in structured exercise programmes influences maternal health, postpartum physical activity levels, and child development over time. Additionally, further research could explore how structured prenatal exercise interventions contribute to long-term behaviour change and family health promotion strategies.

## 5. Conclusions

This study highlights the significant role of social support, group dynamics, and a positive atmosphere in promoting adherence to physical activity during pregnancy. This study advances knowledge in the field by providing an in-depth qualitative exploration of the psychosocial aspects of prenatal exercise, an area that has received less attention compared to the physiological and medical dimensions of physical activity during pregnancy. The holistic, group-based approach not only provided physical benefits but also fostered a sense of community, emotional resilience, and promoted long-term lifestyle changes. Participants reported that the supportive environment, facilitated by both trainer and peer interactions, enhanced their motivation, reduced feelings of isolation, and contributed to their overall well-being during pregnancy and beyond.

Importantly, the long-term impact of the programme was evident, as participants reflected on how their experiences shaped their postpartum behaviours, social connections, and continued physical activity habits.

Given these insights, future prenatal exercise programmes should prioritise social support structures, emotional engagement, and personalised guidance to enhance participation and long-term adherence. To optimise such programmes, future initiatives should consider maintaining small group sizes to enhance peer support and creating a safe and welcoming atmosphere to encourage emotional expression. Involving family members, facilitating postnatal engagement opportunities, and ensuring continuity of social connections beyond the programme may further enhance participation and long-term adherence. Given the limited availability of such comprehensive programmes, expanding access to structured, holistic prenatal exercise opportunities is essential to meet the diverse needs of pregnant women.

Further research should explore more diverse participant samples, including women from different geographical, cultural, and socioeconomic backgrounds, to enhance the representativeness of the findings and better understand the factors influencing adherence in different contexts. Additionally, longitudinal studies are needed to assess the long-term impact of prenatal exercise programmes on maternal health, postpartum physical activity, and family health behaviours. By integrating these considerations, future interventions can be more inclusive, impactful, and sustainable in promoting physical activity among pregnant women.

## Figures and Tables

**Table 1:** Demographic characteristics of the participants (N = 10).

Participant	Age	Education	Pre-Pregnancy Physical Activity Status	Marital Status	Occupation/Workplace
Participant 1	44	Vocational secondary	Active	Married	Pharmacy Technician/Pharmacy
Participant 2	43	Higher	Inactive	Married	Engineer/Own business
Participant 3	34	Higher	Inactive	Married	Clerk/State Water Company
Participant 4	38	Higher	Inactive	Married	Sales Specialist/Phone showroom
Participant 5	40	Higher	Active	Married	Office Worker/Freight forwarding
Participant 6	35	Higher	Inactive	Married	Nutritionist/Private practice
Participant 7	37	Higher	Inactive	Married	Massage therapist/Private office
Participant 8	35	Secondary	Inactive	Unmarried	Parental leave
Participant 9	36	Higher	Inactive	Married	Architect/Online
Participant 10	35	Higher	Active	Married	Geodesist geographer/District office

**Table 2:** Pregnancy-related characteristics of the participants.

Participants	Gestational Week of Last Professional Activity	Sick Leave Status During Pregnancy	Gestational Week Sick Leave Began	Pregnancy Number During Programme Participation	Gestational Week of Programme Enrolment
Participant 1	15	Yes	15	1	20
Participant 2	38	No	-	1	6
Participant 3	20	Yes	20	1	16
Participant 4	9	Yes	9	1	15
Participant 5	36	Yes	37	1	14
Participant 6	9	Yes	9	1	9
Participant 7	20	Yes	20	1	19
Participant 8	14	Yes	15	2	20
Participant 9	36	Yes	-	1	13
Participant 10	38	Yes	24	1	13

**Table 3:** The step-by-step application of Thematic Analysis.

1. Familiarising yourself with your data	The interview recordings were transcribed verbatim (WG; BM), following which, the authors (BM, WG) read and reread the transcripts to become familiar with the breadth and depth of data discussed and initial ideas were noted.
2. Generating initial codes	The authors (BM, WG) created initial codes systematically, on a line-by-line basis, pertinent to the research question. The codes were then collated across the whole data set. In case of discrepancies, a third co-author was consulted (WF-K).
3. Searching for themes	Codes were collated into potential themes (BM, WG).
4. Reviewing themes	Discussion and generation of themes took place via face-to-face and online meetings with the authors. This helped to ensure that themes were relevant to the related coded abstracts and the entire data set (WG, BM). The analytical strategy was data driven and inductive, with a focus on discussing and identifying the principal themes that repeated throughout transcripts. Themes were revised then validated across the data; quotations were chosen to illustrate identified themes.
5. Defining and naming themes	Themes were defined and the overall story of the analysis was drafted (WG, BM, WF-K).
6. Producing the report	The analysis was refined, linking the findings to previous literature and the research question. The broader impact of the findings was considered (BM, WG, WF-K, MP).

## Data Availability

The original contributions presented in this study are included in the article/Supplementary Materials. Further enquiries should be directed to the corresponding author.

## Supplementary Materials

The following supporting information can be downloaded at: https://www.mdpi.com/article/10.3390/healthcare13070727/s1, Table S1: Illustrative Quotes in the psychosocial domain under the core theme of “It’s More than Just Exercise”, Table S2: Consolidated criteria for reporting qualitative studies (COREQ): a 32-item checklist.
