# Supplementary material for: “It’s More than Just Exercise”: Psychosocial Experiences of Women in the Conscious 9 Months Specifically Designed Prenatal Exercise Programme—A Qualitative Study
Source: Healthcare (Basel). 2025 Mar 25;13(7):727. doi: 10.3390/healthcare13070727 (PMC11989166; doi:10.3390/healthcare13070727)
Supplement: Supplementary file 1 [file healthcare-13-00727-s001.zip › healthcare-3483092-supplementary.pdf]

**Table S1**. Illustrative Quotes in the psychosocial domain under the core theme of "It's More Than Just Exercise".

| Themes | Sub-themes | Illustrative Quotes |
|---|---|---|
| Programme as a transformative experience | | *-(….) here, everything was included: exercises, breathing, meeting other pregnant women, and a wonderful atmosphere—you didn't even feel like we were meeting for a fitness class. It felt more like meeting up with friends to laugh, talk, and, additionally, exercise"* ***Participant 5***<br><br>*"Each session was an experience, not just exercise"* ***Participant 8***<br>***"That was the moment when I realised it was more than just exercise. The sessions felt like a ritual, a time to be present, to listen to myself and my body. I started to perceive my needs differently, and I felt that this programme was changing not only my approach to physical activity but also to myself." Participant 10***<br><br>*"During the meetings, it wasn't just about finishing the exercises, but at the same time, also learning - explaining what and how, plus there was counselling too "* ***Participant 2***<br><br>*"Pregnancy was a time of personal growth for me, when (…) I also went to a psychologist for the first time and started learning about my emotions, becoming aware of them. I wasn't connected to my emotions at all before, and I also touched on this in the programme... I feel like there could have been even more of it!"* ***Participant 9*** |
| The Circle of Women – Therapeutic Value of Group Exercise | Overcoming Initial Fears | *"I was afraid whether I'd manage, whether I could do it (…) for example (…) with coordination, you know, during pregnancy—like everyone else going right, and me going left. I also wondered how I'd be perceived, thoughts about myself, like whether I was good enough, or if I'd come across as clumsy or helpless"* ***Participant 10***<br><br>*"I had concerns about whether I'd be able to exercise, whether I'd manage in terms of stamina and breathing"* ***Participant 1***<br><br>***"At first, I wasn't sure if I belonged there, if I would be able to keep up with the others, but when I saw how supportive everyone was, I felt relief. The group gave me confidence and made me feel like I wasn't alone in this journey." Participant 10***<br><br>*"I may have had concerns about my fitness because before pregnancy I didn't lead a particularly active lifestyle and I wasn't the sporty type, so I wondered if I'd be able to keep up with the other women"* ***Participant 4***<br>*"I had the opportunity to participate with other women in the same [life] period as me, with the same problems (...) In fact, we exercised, yes, it was in a sense a physical activity, but above all it was very strengthening for me personally"* ***Participant 8*** |

| | | |
|---|---|---|
| The Circle of Women – Therapeutic Value of Group Exercise | Building Community Through Shared Experiences and Diversity | *"We also knew what might await us in the next stage [of pregnancy], because we were, as far as the group was concerned, each of us was at a different stage of pregnancy"*<br>***Participant 4***<br>*"I found a group of people with whom I lived through this pregnancy, I had friends"*<br>***Participant 4***<br>*"We were treated like a group of friends who know each other, like a sort of mommy family"*<br>***Participant 7***<br><br>*"So well, yes, more of a mental side [was important to me] in a group, because we didn't compete with each other (…) we were well matched"*<br>***Participant 4***<br>*"Well, there was camaraderie between us, no one tried to stand out. Well, we could talk about everything, learn (…) about pregnancy and about being a mother, yes"*<br>***Participant 3***<br>*"[With the trainer] it was a very good, nice collaboration, a nice relationship, without any of that <<I'm the trainer, and you're just here to exercise>> kind of attitude. It was a normal, friendly, peer-like relationship, I would say"*<br>***Participant 1*** |
| The Circle of Women – Therapeutic Value of Group Exercise | The Therapeutic Role of the Group | *"It was also a kind of, you could say, therapy, because it's also that kind of support, a circle of other women who are pregnant. And also the realisation that. Oh, I'm not alone in this. There's one, another, and another who are going through the same thing"*<br>***Participant 10***<br><br>*"It was a circle of women—supporting each other, learning from each other"*<br>***Participant 10***<br><br>***"This is incredibly important for women, especially for first-time mothers, to have a support group and access to such classes. It really brings a sense of calm because pregnancy is a time of both immense joy and, at the same time, stress and a great unknown." Participant 7***<br>*"We were sort of like psychologists, us for each other, each of us exchanging our experiences (…)*<br>***Participant 4***<br>*"We supported each other, we exchanged various experiences with each other and exchanged views, it was, well (…) a support group, where we just supported each other and together we walked this path"*<br>***Participant 4*** |
| The Circle of Women – Therapeutic Value of Group Exercise | Peer Motivation | *"[these classes were a] friendly place, the opposite of the gym. I've never liked the gym or strength training on its own. I preferred group activities, especially in women's clubs—that's where I felt best."*<br>***Participant 4***<br><br>***"The group gave me so much motivation. Seeing other women with similar experiences made me feel like I was in the right*** |

| | | |
|---|---|---|
| | | ***place. When I didn't feel like exercising, I thought of them, and that was enough to get me going."***<br>***Participant 6***<br><br>*"During the classes, I drew motivation from the other women exercising"*<br>***Participant 2***<br><br>***"Sometimes I felt exhausted before the class even started, but seeing the other women getting ready, stretching, chatting—it just made me want to join in. Their energy was contagious, and I never regretted coming."***<br>***Participant 1***<br><br>*"I would leave those classes so energised. Not only because of the endorphins after exercising, but also because of the other women there, all with their bumps just like mine. They were working just as hard, always with a smile on their faces. That was amazing; it was very important and motivating for me"*<br>***Participant 8*** |
| The Circle of Women – Therapeutic Value of Group Exercise | Enhancing Strength Through Group Support | *"First of all, definitely the group—that being in a group gave motivation. When one person went, we all went again, even if sometimes you didn't feel like it, like when you were tired. Toward the end, I didn't always feel like going, but I thought, 'No, I'm going,' because it's something to get out for. And once I went and exercised, everything felt completely different afterward"*<br>***Participant 1***<br><br>***"I never thought that group classes could give me so much strength. It wasn't just about exercising—it was about the people, the energy, and the feeling that we were all in this together."***<br>***Participant 7***<br><br>*"The group was most helpful when we felt physically tired during the exercises and didn't feel like doing it"*<br>***Participant 1***<br><br>*"Sometimes I wanted to just lie down, but I felt that I had to go, like the others do, so I went"*<br>***Participant 8***<br><br>***On some days, I thought, 'Maybe I'll skip today,' but then I'd remember that the others would be there, and I didn't want to miss out. It felt like we were all in this together, pushing through as a team."***<br>***Participant 3***<br><br>*"[The most important thing was] the atmosphere, mainly the atmosphere, because I would get up and go, I just liked it. And then, over time, as my belly grew, I could see that I felt good, and those were the two main driving forces for me: the atmosphere and the fact that I felt really good after those classes"*<br>***Participant 4*** |

| | | |
|---|---|---|
| Atmosphere: Supportive and Empowering Environment | Emotional Climate: Enjoyment and Comfort | *"I just wanted to come to those classes. Sometimes, of course, you'd rather stay at home, maybe skip it—'I'm not going...' because the classes were only two or three times a week. So sometimes I'd think, 'No, I won't go tomorrow...' but then I'd feel like—'Today, I have to'"* <br>***Participant 8***<br>*"The pleasant atmosphere was important. It was motivating, and you could say also informative, because the meetings weren't just about exercising but also about sharing stories and giving each other advice"*<br>***Participant 2***<br>*"After each session, I felt a sense of relief and lightness. It wasn't just the physical effort but also the atmosphere—the warmth, the kindness, the support from the trainer and the other women. It was a safe space where I could be myself." Participant 7*<br>*"The atmosphere was light, fun, and full of joy, which made you want to come back"*<br>***Participant 6***<br>*"Well that very atmosphere was so important, I still smile now when I think about it"*<br>***Participant 8*** |
| Atmosphere: Supportive and Empowering Environment | Freedom to Express Emotions | *"Joy, joy, such a relaxed vibe during the classes. I was eager—I just wanted to go to these classes"*<br>***Participant 7***<br><br>*"It was cheerful. It wasn't like, you know, you weren't allowed to smile. It was genuinely, you could say, all in a pleasant atmosphere"*<br>***Participant 2***<br><br>*"There were jokes in between. Nice, really nice… nice, nice, really nice"*<br>***Participant 7***<br><br>*"There were laughs, and good times—it was more than just exercise"*<br>***Participant 1***<br><br>*"And she [the trainer] would say, 'Now we're doing this, now we're doing that,' and somehow everything was very, very positive. I think Beata was the most positive figure for me; thanks to her, I didn't get discouraged after the first or second session, thinking I couldn't manage. Instead, I always came back with a smile and positive energy because of her"*<br>***Participant 6***<br><br>*"That would have been missing if our trainer hadn't been so infectiously positive with her energy. I think the exercises would have become boring after some time without that. Everything was so well planned, every session was great, and each meeting felt like a really nice experience"*<br>***Participant 8***<br><br>*"She would calm us down, explain everything, and that's what made us feel safe"*<br>***Participant 7*** |

| | | |
|---|---|---|
| | | *"It was also largely thanks to Beata because she knew how to bring everyone together. Of course, she's a professional and focuses on what she does, but at the same time, she was a source of support for us"*<br>***Participant 4***<br>*"What was important was that we were in it together. Also, the relationship with Beata—she supported us so much through it all. That was very important"*<br>***Participant 9*** |
| Atmosphere: Supportive and Empowering Environment | Physical and Structural Elements | *"The music always disarmed us, it energized us to start the workout with a smile on our faces"*<br>***Participant 7***<br><br>***"It was a place where you could truly relax. There was music, it was warm, it was cozy – it wasn't a cold, sterile room, but a space where I genuinely felt good."***<br>***Participant 6***<br><br>*"Beata would also play us relaxing music (…) It gave us a moment to just be with ourselves and also with our little ones. It was like the finishing touch to the sessions. By then, we were a bit tired from the exercises—it wasn't always easy—but when you lay there on the mat or the floor, listening to the music, and she'd say, 'Place your hands on your belly,' it was such a lovely experience. I think those moments really helped to build that bond"*<br>***Participant 10***<br>***"The exercises were very engaging, and various equipment was used, such as balls, resistance bands, and bars, which prevented monotony."***<br>***Participant 9***<br><br>***"The sessions were diverse, never repeating, and included both large and small equipment."***<br>***Participant 3***<br>*"Every exercise session was truly different. Each meeting, even though Beata had her planned repertoire of exercises, was never repetitive. The exercises were varied. We had sessions with exercise balls, sessions at the barre—some of them looked like ballet exercises. We had fun warm-ups inspired by aerobics, exercises with dumbbells and resistance bands, and general conditioning workouts, working the whole body from head to toe. The exercises were adapted to different pregnancy weeks and included standing, lying, and kneeling positions"*<br>***Participant 7*** |
| Atmosphere: Supportive and Empowering Environment | Shared Purpose | *"I think it was the atmosphere during those classes—the shared motivation, the shared experience, preparing for this period and birth—that was the most important"*<br>***Participant 8***<br><br>***"It wasn't just about the exercises, it was the whole experience. The laughter, the casual conversations, the moments of sharing – that was as important as the movement itself. It felt like we were all in this together."***<br>***Participant 3*** |

| | | |
|---|---|---|
| | | *"It strengthened me to see that, you know, everyone had similar problems to some extent, but they always left with a smile and managed to handle everything, especially childbirth"* ***Participant 6***<br><br>***"The atmosphere during the classes was something special. You knew that no matter how you felt that day, someone would understand, we were in it togehter. It was a space where you could just be yourself, and that made all the difference."***<br>***Participant 5***<br><br>*"[The atmosphere] was very positive because it was women who also, you know, sometimes had back pain, or their own pregnancy-related discomforts. But we were going through it together, experiencing it together, preparing for birth together (...) we supported each other. If someone needed to bring something into the room, there was always someone willing to help. It was very, very pleasant"*<br>***Participant 8*** |
| Social Support and Influences Beyond the Programme | Social Support as an Enabler | *"The support of my partner and family was crucial, for example, knowing that I could sometimes leave my daughter with her grandmother. Family support was a significant factor that made it possible for me to take part in this experience"*<br>***Participant 8***<br><br>***"My husband was really supportive. He always encouraged me to go, even when I was feeling tired or unsure. He saw how much better I felt after the classes, and he wanted me to continue because he knew it was good for both me and the baby."***<br>***Participant 5***<br><br>*"My family was very happy with what I was doing and supported me fully. They did everything they could so that I could attend the sessions, right up until the birth—every week or twice a week, depending on how the sessions were scheduled"* ***Participant 7***<br><br>***"The fact that my friends were also pregnant and attended the classes made a huge difference. We would remind each other, check in to see who was coming, and it felt like we were all in this together. That accountability kept me motivated."***<br>***Participant 3*** |
| Social Support and Influences Beyond the Programme | Enduring Social Connections and Sustainable Impact | *"I'm still in touch with the girls; I made friendships that I still maintain, and our kids still play together"*<br>***Participant 6***<br><br>*"I also opened up a bit; those classes with other women, new acquaintances, new friends, then some later get-togethers… Yes, it was all very, very positive and uplifting"*<br>***Participant 8*** |

*"There was a great atmosphere. The women would come, we'd laugh together, and it was such a warm environment. Yes, I miss that"*
***Participant 5***

*"Those were amazing classes back then. I felt great, and during my second pregnancy, I couldn't participate in any classes, and I missed it so much. In my second pregnancy, I had to stay in bed until the end of the third month, and afterward, I had to be very cautious, and I really missed it"*
***Participant 10***

*"I wish those classes were available all the time because, for example, during my second pregnancy, they weren't, and it just wasn't the same"*
***Participant 3***

---

**Table S2. Consolidated criteria for reporting qualitative studies (COREQ): a 32-item checklist.**

| No | Item | Guide questions/description |
|---|---|---|
| **Domain 1: Research team and reflexivity** | | |
| Personal Characteristics | | |
| 1. | Interviewer/facilitator | The interviews were conducted by a psychologist trained in qualitative research, who was not a co-author of the planned study |
| 2. | Credentials | B.M. - PhD, W.G. - MA, W.F.-K. – PhD, M.P. - PhD |
| 3. | Occupation | B.M. – lecturer, researcher, pregnancy exercise specialist; W.G. – lecturer, qualitative researcher, yoga and somatic movement practitioner; W.F.-K. – lecturer, researcher, M.P. – lecturer, researcheradd; |
| 4. | Gender | B.M., W.G., W.F-K. – females, M.P. – male; |
| 5. | Experience and training | What experience or training did the researcher have?<br>B.M.– Eight years experience in pregnancy-related research, thirteen years experience as a pregnancy exercise specialist and seventeen years experience as an athletics trainer; W.G – Phd candidate, seven years experience in qualitative methodologies; W.F. – 13 years of experience in pregnancy-related research, university professor; M.P. specialist physiotherapist, university professor; |
| Relationship with participants | | |
| 6. | Relationship established | The first author of the planned study has a previous relationship with the participants as an exercise trainer. The remaining research team members do not have any previous relationship with the participants. |
| 7. | Participant knowledge of the interviewer | None of the participants had any prior personal acquaintance with the interviewer before the commencement of the interviews. This lack of pre-existing relationship helped to establish a neutral context for the discussions, minimizing the potential for bias related to familiarity or shared history. It also ensured that participants approached the interview process without preconceived notions |

| No | Item | Guide questions/description |
| --- | --- | --- |
| | | about the interviewer, allowing for more open and authentic responses. |
| 8. | Interviewer characteristics | The interviewer is a psychologist trained in qualitative research methods. To mitigate potential bias, the research team decided that the interviews would not be conducted by the first author of the study, given her prior relationship with the participants. Instead, a neutral psychologist with extensive training and experience in communication and active listening skills was selected. This interviewer, like all participants, is Polish and shares a cultural and linguistic background with them. Additionally, the interviewer has a professional and personal interest in pregnancy and health-related topics, as she works directly with pregnant women in a local hospital, further enhancing her understanding of the subject matter. |
| **Domain 2: study design** | | |
| Theoretical framework | | |
| 9. | Methodological orientation and Theory | The study employed a qualitative methodological orientation, using in-depth semi-structured interviews to gather rich, detailed accounts of participants' experiences and perspectives. Thematic analysis was chosen as the analytical approach, allowing for the identification, analysis, and interpretation of patterns and themes within the data. This approach is grounded in a constructivist paradigm, which acknowledges that knowledge and meaning are co-constructed through interaction between the researcher and participants. |
| Participantselection | | |
| 10. | Sampling | Purposive sampling. |
| 11. | Method of approach | Data were collected through semi-structured interviews and analysed using Thematic Analysis to identify patterns and themes in participants' narratives.The interviews were arranged via telephone to schedule a specific date (day, time, location), and were subsequently conducted in person. |
| 12. | Samplesize | 10. |
| 13. | Non-participation | 2 out of 12 participants could not be reached via phone. |

| No | Item | Guide questions/description |
|---|---|---|
| Setting | | |
| 14. | Setting of data collection | Movement Studio belonging to the University. |
| 15. | Presence of non-participants | None. |
| 16. | Description of sample | Women who completed the "Conscious nine months" exercise programme during their pregnancy in the years 2017-2019. |
| Data collection | | |
| 17. | Interview guide | An interview guide with questions and prompts is provided as a supplementary file. The pilot study has been conducted. |
| 18. | Repeatinterviews | No repeat interviews have been conducted. |
| 19. | Audio/visualrecording | The data were audio recorded. |
| 20. | Field notes | Field notes were taken and recorded in real-time during and after the interviews. |
| 21. | Duration | 47-65 mins. |
| 22. | Data saturation | Data saturation was achieved after conducting 8 interviews, as no new themes, codes, or insights emerged during the analysis of the subsequent 2 interviews. |
| 23. | Transcripts returned | The transcripts were returned to participants for comment and/or correction. |
| **Domain 3: analysis and findings**z | | |
| Data analysis | | |
| 24. | Number of data coders | Two coders working independently (B.M. and W.G.) and two other research members (W.F.-K. and M.P.) were involved in case of doubts/problems. |
| 25. | Description of the coding tree | A coding tree was developed to guide the thematic analysis, organizing the data into three main themes and multiple sub-themes. While a visual representation of the coding tree is not included in this manuscript, a detailed description of the themes and sub-themes is provided in the results section. |

| No | Item | Guide questions/description |
|---|---|---|
| 26. | Derivation of themes | Inductive approach: Themes emerged directly from the data without being pre-determined by existing theories or frameworks. |
| 27. | Software | None used. |
| 28. | Participant checking | Participants were asked to provide feedback on the findings. |
| Reporting | | |
| 29. | Quotations presented | Participant quotations were presented to illustrate the themes and findings (table provided as a supplementary file). Each quotation was identified by the participant number. |
| 30. | Data and findings consistent | The findings are consistently grounded in the data, with direct quotes from participants provided throughout the results section to illustrate each theme. These quotes were selected to represent the breadth and depth of participant perspectives and ensure that the themes accurately reflect the data. |
| 31. | Clarity of major themes | The study clearly identifies and describes three major themes: (1) *The Circle of Women – Therapeutic Value of Group Exercise*, (2) *Atmosphere: Supportive and Empowering Environment*, and (3) *Social Support and Influences Beyond the Programme*. Each theme is thoroughly defined in the results section, reflecting distinct yet interconnected aspects of participants' experiences. For example, *The Circle of Women* explores how participants overcame initial fears, built community, and drew strength and motivation from group support. *Atmosphere* highlights the role of emotional climate, the trainer's influence, and structural elements in creating a positive and empowering environment. *Social Support and Influences Beyond the Programme* emphasizes the enduring social connections and sustainable impact of the program. Each major theme is presented with detailed descriptions and direct participant quotes to ensure clarity and relevance. |
| 32. | Clarity of minor themes | Minor themes are clearly identified and described as they contribute depth and nuance to the major themes. For example, within *The Circle of Women*, minor themes such as *Overcoming Initial Fears* and *Building Community Through Shared Experiences and Diversity* illustrate the process of group bonding and individual growth. Similarly, within *Atmosphere*, minor themes such as *Freedom to Express Emotions* |

| No | Item | Guide questions/description |
| --- | --- | --- |
| | | and *Physical and Structural Elements* highlight specific aspects of the supportive environment, such as the role of space and freedom in creating a positive experience. Lastly, within *Social Support and Influences Beyond the Programme*, *Social Support as an Enabler* and *Enduring Social Connections* underscore the broader and long-term impact of the program. These minor themes are presented alongside major themes in the results, supported by direct participant quotes to ensure coherence and transparency in the findings. |
